# On the Inhalation of Sulphuric Ether

**Published:** 1847-01

**Authors:** J. B. Flagg

**Affiliations:** Philadelphia, Surgeon Dentist


*On the Inhalation of Sulphuric Ether.* By J. B. Flagg, M. D. of Philadelphia, Surgeon Dentist.

The medical world is pretty fully advised as to the narcotic influence of this drug, when its vapour is inhaled into the lungs *under peculiar circumstances.*

Desirous of promoting the cause of science, and curious to test its efficacy when applied to painful operations, I have been engaged for several weeks past in experimenting with it, both in its pure washed state, and compounded to a very limited extent with oil of wine; first upon myself, and then upon many friends. I will proceed to give a few remarks as to its legitimate use, and its recent *illegitimate introduction* to the profession of surgery.

The effects of sulphuric ether, when inhaled, have long been known to be similar to those of nitrous oxide; varying, according to idiosyncrasy and to the object in view. If inhaled with a certain degree of doubt as to its capacity to effect, it is most sure to produce a melancholy train of thought for the time being, accompanied with weeping or other symptoms of distress. If taken with a view to produce merriment, its tendency, in a large proportion of cases, is to induce pleasurable sensations, as evinced by laughter, dancing, grotesque gestures, &c. When taken for the purpose of submitting to an operation, the mind of the patient, having dwelt much upon the ultimate object to be attained, is calmed and trained to that perfect state of inactivity, which allows the operation to proceed with but slight, if any, disposition to resist, on the part of the patient.

The cerebrum, while under its influence, acts from exciting causes, similar to those which occur frequently during natural sleep; that is, *external circumstances will suggest the idea.* Two cases will serve to illustrate:

Miss F., a young lady about 18 years of age, of nervous temperament, had two teeth removed after two minutes inhaling the vapour; she was at first perfectly quiet, opened the mouth at my request, and allowed the instrument (forceps) to be placed upon the tooth; but as it was necessary to force them well under the alveoli, she made considerable resistance, and appeared to suffer much; she screamed loudly, saying, "*stop pulling.*" In less than two minutes after the teeth were removed, and when the effect had entirely passed off, she assured her mother that she had not been in the least hurt. Upon being questioned as to why she manifested so much distress, she replied that her dream was an unpleasant one; that she thought she was on board a vessel, and perceiving that they were going upon the rocks, she called to the man at the helm to "*stop pulling!*" supposing that the vessel was propelled by this means. Now as the words made use of were a natural outcry, I prefer to attribute to *them* the suggestion of the dream, than to marvel at the coincidence.

A young man, æt. 20, was desirous of having a painful *molar* of the lower jaw extracted; his doubts were very great as to the possibility of destroying sensation; but still desired the experiment. For this operation I used the German Key; he manifested considerable pain; carried his hand to the mouth; swayed the body forward and back, as in much agony. I asked if it hurt him much; he said, "*indeed it did.*" I then requested him to tell me if the tooth was out; he said, "*no.*" Here I made the remark that it was a humbug; he immediately replied that "*it was a humbug*"—that "he did not believe it could be done"—"expected to be imposed upon when he came." At this point he recovered consciousness; smiled when I showed him his tooth; assured me that he suffered *no pain whatever.* Upon my asking him why he called it a humbug, he denied having said so; clearly illustrating that his ideas for the moment were dictated by my method of treating the matter.

From various experiments, I am satisfied that it is by no means necessary to produce *entire unconsciousness,* in order to act sufficiently upon the sensorium to allow of any operation being performed, of not more than two or three minutes duration. Several recent cases have been so treated by me that my patients were perfectly aware of their position; having a full knowledge of every process in the operation; and expressing themselves, invariably, as much delighted with the result; one stating that "*it felt like extracting the tooth from a block of wood.*"

The medical properties and uses of sulphuric ether are well described in Wood and Bache's Dispensatory, page 810: "A powerful diffusible stimulant, though transient in its operation." "It is also esteemed anti-spasmodic and narcotic." "Its vapour, when breathed, produces a transient intoxication, resembling that caused by respiring *nitrous oxide*, but dangerous if carried too far." "It is beneficial in some stages of low fevers; likewise good in nervous headache, unattended with *vascular fulness*—some stages of hysteria; and generally in nervous and painful diseases, which are unaccompanied by inflammation." "In catarrhal dyspnœa and spasmodic asthma, its vapour may be inhaled with advantage. In nausea it is given as a cordial; likewise in cramp in the stomach, &c.

From this abbreviated quotation from the above-mentioned work, it will be seen that the medicinal virtues of the article under consideration, have been well understood by the profession for a long time; and that it is esteemed an agent of so much potency, as to be used with much caution, even by the faculty; hence I am at a loss to comprehend the validity of a certificate, emanating from the hands of *one* quack authorising another of similar pretensions to administer it under any circumstances whatever. And should any accident occur in this view of the case, I trust that the *medical* and *legal* world will know how to discriminate.

I would not be understood as speaking lightly of all those gentlemen engaged in the dental art, who may not be provided with medical diplomas. There are many such, whose work show them to be highly deserving of public confidence; and it is my belief that all who may be desirous of employing this agent, will be so led in their studies, physiological and pathological, that they will sooner or later demand and receive such testimonials as must serve to elevate our much abused and down-trodden profession.

It is much to be hoped that some attention may be bestowed upon this subject by our medical colleges: suffering humanity demands that the whole question be properly investigated and put upon such a footing as will secure to the community what Dr. Bigelow, in his article of the 18th Nov., Boston Med. and Surg. Journal, seems so desirous of accomplishing—A SUITABLE GUARANTEE AGAINST ITS ABUSE.

